# 

**Published:** 1887-02

**Authors:** 


					﻿BOOKS AND PAMPHLETS RECEIVED.
Fifty-seventh Annual Report of Board of Mana-
gers of Philadelphia Lying-in Charity.
Inter-State Notification ; Its principles demonstrated
in the history of yellow fever at Biloxi, Miss.
A Treatise on Some Diseases of the Bones and Lig-
aments of the Spine. S. M. Cate, M. D.
Address on Relation of Quarantine to Shipping
Interests. By Joseph Holt, M. D.
Hereditary Insanity. By S. Lilienthal, M. D.
NOTICE.
.4W articles for publication, books for review, business .communications, etc.,
must be sent to Editors, 1123 Spruce Street, Philadelphia.
Subscribers will please notice that this Journal will be sent until arrears
are paid- and it is ordered discontinued.
W* Subscribers, Contributors, and Exchanges,
please TAKE NOTICE. The Office of THIS
JOURNAL has been removed to 1123 Spruce
■ Street, where all communications must be sent.
All subscriptions are due in advance; subscribers will therefore
please forward their subscriptions at once; let each subscriber see
that his account is settled promptly.
Subscribers are respectfully requested to make all checks
and money-orders payable to THE HOMOEOPATHIC
PHYSICIAN, ja/nd not to either of the Editors.
				

